# Depression and Anxiety in Melanoma Patients and Their Relationships with Dermatology-Related Quality of Life: A Prospective Observational Cross-Sectional Study

**DOI:** 10.3390/jcm15197524

**Published:** 2026-09-27

**Authors:** Maja Hrynczyszyn, Michalina Świetlicka, Paulina Piesch, Maciej Krężel, Hanna Adamska, Inga Buława, Marta Szepietowska, Piotr K. Krajewski, Przemysław Pacan, Grażyna Kamińska-Winciorek, Marcin Ziętek, Łukasz Matusiak, Jacek C. Szepietowski

**Affiliations:** 1 Students’ Research Group, Division of Dermatology, Venereology and Clinical Immunology, Faculty of Medicine, Wroclaw University of Science and Technology, 50-377 Wroclaw, Poland276241@student.pwr.edu.pl (H.A.);; 2 Doctoral School of Medical and Health Sciences, Jagiellonian University Medical College, 31-530 Cracow, Poland; marta.szepietowska@doctoral.uj.edu.pl; 3 Department of Dermatology, Jagiellonian University Medical College, 31-503 Cracow, Poland; 4 Division of Dermatology, Venereology and Clinical Immunology, Faculty of Medicine, Wroclaw University of Science and Technology, 51-377 Wroclaw, Poland; piotr.krajewski@pwr.edu.pl (P.K.K.); lukasz.matusiak@pwr.edu.pl (Ł.M.); 5 Department of Dermato-Venereology, 4th Military Hospital, 50-981 Wroclaw, Poland; 6 Department of Psychiatry, Institute of Medical Sciences, Rzeszow University, 35-959 Rzeszow, Poland; ppacan@ur.edu.pl; 7 Skin Cancer and Melanoma Team, Department of Bone Marrow Transplantation and Haematology-Oncology, Maria Sklodowska-Curie National Research Institute of Oncology, 44-102 Gliwice Branch, Poland; dermatolog.pl@gmail.com; 8 Lower Silesian Oncology, Pulmonology and Hematology Center, 53-413 Wroclaw, Poland; marcin.zietek@umw.edu.pl; 9 Department of Oncology, Faculty of Medicine, Wroclaw Medical University, 53-413 Wroclaw, Poland

**Keywords:** melanoma, depression, anxiety, quality of life, psycho-oncology

## Abstract

**Background/Objectives:** Patients with melanoma may experience depression and anxiety, but their relationship with dermatology-related quality of life (QoL) remains insufficiently characterized. This study assessed depressive and anxiety symptoms and their associations with QoL, sex, disease stage, and time since diagnosis. **Methods:** This cross-sectional study included 162 adults with histologically confirmed melanoma recruited consecutively at two Polish tertiary oncology centers. Participants completed the Patient Health Questionnaire-9 (PHQ-9), Generalized Anxiety Disorder-7 (GAD-7), Hospital Anxiety and Depression Scale (HADS), and Dermatology Life Quality Index (DLQI). Associations were examined using non-parametric tests and multivariable linear regression. **Results:** Positive screening rates were 8.8% for depression using PHQ-9 and 15.2% using HADS-D, and 19.1% for anxiety using GAD-7 and 27.4% using HADS-A. Women had higher PHQ-9, GAD-7, and HADS-A scores than men. All psychological measures correlated with DLQI scores (Spearman’s ρ = 0.328–0.414; all adjusted *q* < 0.001). In multivariable models, higher DLQI scores independently correlated with greater symptom severity across all four measures (all *p* ≤ 0.001). Female sex independently correlated with higher PHQ-9 and GAD-7 scores, while younger age correlated with higher PHQ-9, GAD-7, and HADS-A scores. Neither melanoma stage nor time since diagnosis was independently associated with psychological symptoms. **Conclusions:** Depressive and anxiety symptoms are clinically relevant in melanoma and closely associated with poorer dermatology-related QoL. Psychological screening should not be limited to patients with advanced disease. Females and younger patients may warrant particular attention.

## 1. Introduction

Melanoma is a malignant neoplasm arising from melanocytes and represents one of the most clinically aggressive forms of skin cancer. Its incidence has increased substantially over recent decades, particularly in populations of European ancestry [1,2,3]. According to GLOBOCAN 2022 estimates, approximately 332,000 new cases of melanoma and nearly 59,000 melanoma-related deaths occurred worldwide in 2022 [4]. Although earlier detection and major advances in systemic treatment have considerably improved outcomes for many patients, melanoma remains a potentially life-threatening disease characterized by a highly stage-dependent prognosis and a risk of recurrence or progression. Consequently, an increasing number of patients live for prolonged periods with the diagnosis and require long-term surveillance, making the psychological and quality of life (QoL) consequences of melanoma increasingly relevant components of comprehensive care [5,6].

Among the psychological consequences of melanoma, depressive and anxiety symptoms have received particular attention. The diagnosis of a potentially life-threatening malignancy, uncertainty regarding prognosis and recurrence, repeated follow-up examinations, and—in patients with advanced disease—the burden of systemic treatment may all contribute to persistent emotional distress. Recent systematic reviews and meta-analyses reported increased prevalence of depressive and anxiety symptoms in melanoma subjects, which varied throughout the disease trajectory [7,8]. These data emphasize that estimates of psychological morbidity in melanoma depend considerably on the clinical setting, time since diagnosis, treatment status, and instruments used for assessment.

Importantly, psychological distress in melanoma does not appear to be determined solely by objective oncological severity. The 2024 meta-analysis identified female sex, younger age, and lower educational level as factors associated with a higher likelihood of anxiety, while more advanced disease was associated with greater depressive burden [4]. However, longitudinal observations indicate a more complex pattern. In a 4-year study of patients with high-risk primary cutaneous melanoma, melanoma-related anxiety and depression decreased substantially during the first months following diagnosis, whereas a subgroup of patients developed persistent or newly emerging psychological symptoms [5]. More recent prospective evidence also suggests that psychological burden may be substantial even in patients with relatively early-stage melanoma. In a 2025 cohort of working-age patients with stages 0–IIIC melanoma, impaired general well-being was observed in a considerable proportion of patients irrespective of tumor stage, while women demonstrated lower health-related quality of life (HRQoL) and a greater need for psycho-oncological support [9]. Thus, clinical stage alone may not adequately identify patients at risk of psychological morbidity.

Depression and anxiety are also closely intertwined with patients’ perception of their health and everyday functioning [10]. QoL is a multidimensional outcome encompassing physical, emotional, social, and functional domains and has become increasingly important in melanoma survivorship research. In a large register-based study of 689 long-term melanoma survivors, depressive and anxiety symptoms remained clinically relevant years after diagnosis and were closely related to factors associated with poorer QoL, including fear of recurrence, insufficient social support, pessimism, and maladaptive coping [11]. Studies conducted shortly after melanoma diagnosis have likewise demonstrated meaningful supportive-care and psychological needs even among patients with localized disease [12]. More recently, prospective patient-reported outcome studies have further highlighted substantial interindividual variability in HRQoL trajectories following melanoma diagnosis and the potential value of repeated psychological and QoL assessment [9]. Nevertheless, many previous melanoma studies have primarily employed generic cancer-related or melanoma-specific HRQoL instruments, whereas the relationship between depressive and anxiety symptoms and dermatology-related QoL has received comparatively less attention. Furthermore, differences between psychological screening instruments may substantially influence estimates of depressive and anxiety symptom burden. The use of complementary measures may therefore provide a broader characterization of psychological status than reliance on a single questionnaire [10].

The primary aim of the present study was therefore to assess the prevalence and severity of depressive and anxiety symptoms in patients with melanoma and to evaluate their associations with dermatology-related QoL. Additionally, we investigated whether psychological symptom severity was associated with sex, melanoma stage, and time since diagnosis.

## 2. Materials and Methods

### 2.1. Study Design, Setting, Participants, and Ethics

This observational cross-sectional study was conducted between June 2025 and February 2026 at two tertiary oncology centers in Poland: the Maria Skłodowska-Curie National Research Institute of Oncology, Gliwice Branch, and the Lower Silesian Oncology, Pulmonology and Hematology Center in Wroclaw. Consecutive adult patients with histologically confirmed melanoma attending routine outpatient follow-up visits were invited to participate in the study.

A total of 191 patients were approached. Of these, 162 provided written informed consent and completed required assessments and were therefore included in the final analysis, corresponding to a response rate of 84.8%. The study population comprised 67 women (41.4%) and 95 men (58.6%), with a mean age of 59.8 ± 13.7 years (range: 29–92 years). Demographic and clinical information was collected using a structured questionnaire and review of available medical documentation. The recorded variables included age, sex, time since melanoma diagnosis, and clinical stage of the disease (Table 1).

The present analysis was based on the same patient cohort as our previously published study on illness acceptance, which addressed a distinct research question [13]. The present study specifically focused on depressive and anxiety symptoms and their associations with dermatology-related quality of life (QoL). For this particular study participants completed validated Polish-language self-administered questionnaires evaluating depressive and anxiety symptoms and dermatology-related QoL.

The study was conducted in accordance with the principles of the Declaration of Helsinki. The study protocol was approved by the Ethics Committee of the Lower Silesian Chamber of Physicians and Dentists in Wroclaw, Poland (decision no. 13/BNBO/2025). All participants provided written informed consent before inclusion in the study.

### 2.2. Clinical Assessment of Melanoma

Melanoma stage was determined according to the eighth edition of the American Joint Committee on Cancer (AJCC) staging system [14], on the basis of the characteristics of the primary tumour, regional lymph node involvement and the presence of distant metastases. Patients were classified as having stage I, II, III or IV disease. For the purposes of the present analyses, AJCC stage was treated as an indicator of objective disease advancement and was examined in relation to depressive symptoms, anxiety symptoms and dermatology-related QoL.

### 2.3. Assessment of Depression and Anxiety

Depressive and anxiety symptoms were assessed using three validated Polish-language self-administered instruments: the Patient Health Questionnaire-9 (PHQ-9) [15,16], the Generalized Anxiety Disorder-7 (GAD-7) [17,18], and the Hospital Anxiety and Depression Scale (HADS) [19]. Two independent measures were therefore available for both depression (PHQ-9 and HADS-D) [15,16,18] and anxiety (GAD-7 and HADS-A) [17,18,19].

#### 2.3.1. Patient Health Questionnaire-9

The PHQ-9 was used to assess the presence and severity of depressive symptoms during the preceding two weeks [15]. It consists of nine items corresponding to the diagnostic symptoms of major depressive disorder. Each item is rated from 0 (“not at all”) to 3 points (“nearly every day”), resulting in a total score ranging from 0 to 27 points. Scores of 0–4 points indicate minimal or absent depressive symptoms, whereas scores of 5–9, 10–14, 15–19, and 20–27 points indicate mild, moderate, moderately severe, and severe depressive symptoms, respectively. A PHQ-9 score of ≥10 was considered indicative of clinically relevant depressive symptoms and was used as the threshold for a positive depression screen [15,16].

#### 2.3.2. Generalized Anxiety Disorder-7

The GAD-7 was used to evaluate anxiety symptoms experienced during the preceding two weeks [17]. The questionnaire comprises seven items, each scored from 0 (“not at all”) to 3 points (“nearly every day”), yielding a total score between 0 and 21 points. Scores of 0–4, 5–9, 10–14, and 15–21 points correspond to minimal, mild, moderate, and severe anxiety symptoms, respectively. A score of ≥8 points was considered indicative of clinically relevant anxiety symptoms and was used as the threshold for a positive anxiety screen [17,18]. Severity categories are reported separately using the conventional 5/10/15-point boundaries; the screening threshold and the severity categories are therefore not interchangeable, and the proportion of patients screening positive is not equal to the proportion in the moderate and severe categories combined.

#### 2.3.3. Hospital Anxiety and Depression Scale

The Hospital Anxiety and Depression Scale was additionally used to assess psychological distress [19]. The instrument was originally developed for use in patients with somatic diseases and limits the contribution of physical symptoms that may overlap with manifestations of the underlying medical condition. The scale comprises two seven-item subscales assessing anxiety (HADS-A) and depression (HADS-D). Each item is scored from 0 to 3 points, resulting in scores ranging from 0 to 21 points for each subscale. Scores of 0–7 points were considered within the normal range, 8–10 points were regarded as borderline, and scores of ≥11 points indicated clinically relevant depressive or anxiety symptoms [ref]. For prevalence analyses, a cut-off of ≥8 was used to identify patients screening positive for at least borderline depressive or anxiety symptoms [19].

The simultaneous use of PHQ-9/GAD-7 and HADS was intended to provide complementary assessment of psychological distress. PHQ-9 and GAD-7 allow detailed grading of depressive and anxiety symptom severity, whereas HADS was specifically developed for populations with somatic illness and reduces the potential influence of disease-related physical symptoms on psychological assessment.

### 2.4. Assessment of Dermatology-Related Quality of Life

Dermatology-related QoL was assessed using the validated Polish version of the Dermatology Life Quality Index (DLQI) [20]. The DLQI consists of 10 questions assessing the impact of a dermatological condition on symptoms and feelings, daily activities, leisure, work or education, personal relationships, and treatment during the preceding seven days. Each item is scored from 0 to 3 points, producing a total score ranging from 0 to 30 points, with higher scores indicating greater impairment of QoL [20].

### 2.5. Data Completeness and Handling of Missing Data

Total scores were computed only for participants who had completed all items of the given instrument; missing items were not imputed and were never replaced by zero. Therefore, the number of available observations differs between instruments and is reported alongside every result.

### 2.6. Statistical Analysis

Statistical analyses were performed using IBM SPSS Statistics version 26.0 (IBM Corp., Armonk, NY, USA) and Python version 3.11 (SciPy 3.11.0, statsmodels). Normality was assessed using the Shapiro–Wilk test; all scale scores departed from normality (*p* < 0.001), and non-parametric methods were therefore used throughout. Descriptive statistics are presented as mean ± SD and as median with interquartile range (IQR); strongly right-skewed variables are reported as medians with IQRs. Males and females were compared using the Mann–Whitney U test and Fisher’s exact test, AJCC stages using the Kruskal–Wallis test, and continuous variables were correlated using Spearman’s ρ, with *p* values for the DLQI correlations adjusted by the Benjamini–Hochberg procedure (reported as q).

Multivariable linear regression was used to identify independent correlates of psychological symptom severity. Four models were fitted, with the PHQ-9, HADS-D, GAD-7 and HADS-A total scores as dependent variables; each included the DLQI total score, sex, age, log-transformed time since diagnosis and AJCC stage (ordinal, I–IV), entered simultaneously without stepwise selection. Results are reported as unstandardised (B) and standardised (β) coefficients with 95% confidence intervals, and model fit as R, R^2^ and adjusted R^2^. Multicollinearity, homoscedasticity and independence of residuals were verified using variance inflation factors, the Breusch–Pagan test and the Durbin–Watson statistic; because residuals deviated from normality, models were refitted with heteroscedasticity-consistent (HC3) standard errors and bootstrapped confidence intervals (10,000 resamples). Sensitivity analyses used stage as indicator variables, omitted age, applied a square-root transformation to the dependent variables, and reversed the model direction with the DLQI as the dependent variable. Because the four dependent variables were strongly intercorrelated, a multivariate analysis of covariance with all four total scores entered jointly and the same covariates was additionally performed as an omnibus test, with results reported as Pillai’s trace. All tests were two-sided and *p* < 0.05 was considered significant.

## 3. Results

### 3.1. Depression

The mean PHQ-9 score in the study population was 4.16 ± 4.03 points (median 3, IQR 1–6; n = 148). Using a PHQ-9 score of ≥ 10 as the threshold for clinically relevant depressive symptoms, 8.8% of patients (n = 13) screened positive for depression. According to the established PHQ-9 severity categories, 91 patients (61.5%) had minimal or no depressive symptoms, 44 (29.7%) had mild symptoms, 8 (5.4%) had moderate symptoms and 5 (3.4%) had moderately severe symptoms; none of the participants met the criteria for severe depressive symptoms (Figure 1a).

The mean HADS-D score was 3.93 ± 3.47 points (median 3, IQR 1–6; n = 158). Scores in the normal range (0–7) were recorded in 134 patients (84.8%), borderline scores (8–10) in 12 (7.6%) and clinically relevant scores (≥ 11) in 12 (7.6%); using the ≥ 8 cut-off, 24 patients (15.2%) screened positive for at least borderline depressive symptoms.

Female patients demonstrated significantly higher PHQ-9 scores than males (5.29 ± 4.28 vs. 3.42 ± 3.69 points; *p* = 0.002), whereas no significant sex difference was observed for HADS-D scores (4.42 ± 3.76 vs. 3.58 ± 3.23 points; *p* = 0.180) (Table 2, Figure 2). The proportion of positive depression screens did not differ significantly between women and men for either instrument (PHQ-9 ≥ 10: 11.9% vs. 6.7%, *p* = 0.375; HADS-D ≥ 8: 21.2% vs. 10.9%, *p* = 0.114).

### 3.2. Anxiety

The mean GAD-7 score was 3.84 ± 4.22 points (median 3, IQR 0–6; n = 152). Clinically relevant anxiety symptoms, defined as a GAD-7 score ≥ 8, were present in 19.1% of patients (n = 29). According to GAD-7 severity categories, 105 patients (69.1%) had minimal or no anxiety symptoms, 32 (21.1%) had mild symptoms, 9 (5.9%) had moderate symptoms, and 6 (3.9%) had severe symptoms (Figure 1b).

The mean HADS-A score was 5.43 ± 3.61 points (median 5, IQR 3–8; n = 157). Scores were within the normal range in 114 patients (72.6%), borderline in 27 (17.2%) and clinically relevant in 16 (10.2%); 43 patients (27.4%) reached the ≥ 8 cut-off. Anxiety was thus the more prevalent of the two psychological outcomes assessed, irrespective of the instrument used.

Anxiety symptoms differed significantly according to sex. Female patients had higher GAD-7 scores than males (5.44 ± 4.68 vs. 2.74 ± 3.50 points; *p* < 0.001) and higher HADS-A scores (6.26 ± 4.08 vs. 4.82 ± 3.12 points; *p* = 0.046) (Table 2, Figure 2). The difference was also apparent at the level of positive screens: 32.3% of women versus 10.0% of men screened positive on the GAD-7 (*p* = 0.001) and 36.4% versus 20.9% on the HADS-A (*p* = 0.046).

### 3.3. Depression and Anxiety in Relation to Melanoma Stage, Disease Duration and Age of Patients

Neither depressive nor anxiety symptom severity was significantly associated with melanoma stage. Scores did not differ across AJCC stages I–IV for the PHQ-9 (*p* = 0.749), HADS-D (*p* = 0.530), GAD-7 (*p* = 0.925) or HADS-A (*p* = 0.590). Likewise, no significant correlations were identified between time since melanoma diagnosis and any of the four psychological measures (ρ from −0.045 to +0.127; all *p* > 0.13).

In contrast, younger age was consistently associated with greater psychological symptom burden: age correlated inversely with GAD-7 (ρ = −0.292; *p* = 0.001), HADS-A (ρ = −0.264; *p* = 0.002) and PHQ-9 scores (ρ = −0.225; *p* = 0.010), with a non-significant trend for HADS-D (ρ = −0.145; *p* = 0.088).

### 3.4. Relationships Between Depression, Anxiety, and Dermatology-Related Quality of Life

Higher levels of depressive symptoms were significantly associated with greater impairment of dermatology-related QoL. PHQ-9 scores correlated positively with DLQI total scores (ρ = 0.377; *p* < 0.001; n = 136) (Figure 3a) and HADS-D scores showed a comparable association (ρ = 0.411; *p* < 0.001; n = 144) (Figure 3b).

A similar relationship was observed for anxiety. GAD-7 scores correlated positively with DLQI scores (ρ = 0.328; *p* < 0.001; n = 139) (Figure 3c), as did HADS-A scores (ρ = 0.414; *p* < 0.001; n = 143) (Figure 3d), indicating progressively greater QoL impairment with increasing severity of anxiety symptoms. All four associations remained significant after Benjamini–Hochberg correction for multiple testing (all q < 0.001).

As expected, depressive and anxiety symptoms were strongly interrelated. PHQ-9 scores correlated with GAD-7 scores (ρ = 0.745; *p* < 0.001) and HADS-D scores with HADS-A scores (ρ = 0.613; *p* < 0.001). Agreement between the two families of instruments was also substantial (PHQ-9 vs. HADS-D r = 0.642; GAD-7 vs. HADS-A ρ = 0.695; both *p* < 0.001).

### 3.5. Independent Predictors of Depressive and Anxiety Symptoms

In multivariable linear regression, greater impairment of dermatology-related QoL was an independent correlate of both depressive and anxiety symptom severity, consistently across all four instruments. Each additional DLQI point corresponded to an increase of 0.476 PHQ-9 points (95% CI 0.316–0.636; β = 0.477; *p* < 0.001), 0.419 HADS-D points (95% CI 0.274–0.564; β = 0.468; *p* < 0.001), 0.321 GAD-7 points (95% CI 0.139–0.504; β = 0.295; *p* = 0.001) and 0.376 HADS-A points (95% CI 0.217–0.536; β = 0.383; *p* < 0.001). In every model the DLQI was the strongest or second strongest predictor (Table 3).

Female sex remained independently associated with higher PHQ-9 (B = 1.826; 95% CI 0.615–3.038; *p* = 0.004) and GAD-7 scores (B = 2.263; 95% CI 0.891–3.636; *p* = 0.002) after adjustment, whereas the association with HADS-A was of borderline significance (B = 1.154; 95% CI −0.013 to 2.322; *p* = 0.053) and no independent effect was observed for HADS-D (*p* = 0.110). Younger age was independently associated with greater anxiety (GAD-7: B = −0.090 per year; 95% CI −0.140 to −0.039; *p* = 0.001; HADS-A: B = −0.072; 95% CI −0.115 to −0.029; *p* = 0.001) and with higher PHQ-9 scores (B = −0.052; 95% CI −0.095 to −0.008; *p* = 0.020).

Neither AJCC stage nor time since diagnosis was an independent predictor of any of the four psychological measures (all *p* > 0.27). The models explained 27.2–35.0% of the variance in psychological symptom severity (adjusted R^2^ 0.240–0.319; all models *p* < 0.001).

Because the four outcome measures were strongly intercorrelated (ρ = 0.61–0.75), a multivariate analysis of covariance with all four total scores entered jointly as dependent variables and the same set of covariates was performed as an omnibus test (n = 107, complete data for all four instruments). This confirmed the univariate results: the DLQI (Pillai’s trace = 0.331; F(4,98) = 12.11; *p* < 0.001), age (0.117; F(4,98) = 3.24; *p* = 0.015) and female sex (0.113; F(4,98) = 3.11; *p* = 0.019) were significantly associated with the set of psychological outcomes, whereas AJCC stage (*p* = 0.846) and time since diagnosis (*p* = 0.544) were not.

## 4. Discussion

The present study provides a comprehensive assessment of depressive and anxiety symptoms in patients with melanoma and demonstrates their close relationship with dermatology-related QoL. Several findings deserve particular attention. First, clinically relevant depressive symptoms were identified in approximately one in ten patients according to the PHQ-9, while even more patients were screened positive for clinically relevant anxiety according to the GAD-7. Second, women demonstrated a consistently greater psychological burden, particularly with respect to anxiety. Third, neither depressive nor anxiety symptom severity was significantly associated with melanoma stage or time since diagnosis. Finally, and importantly, all four psychological measures used in the study showed significant positive correlations with DLQI, indicating that greater depression and anxiety symptom burden was consistently associated with poorer dermatology-related QoL.

The prevalence of psychological symptoms observed in our cohort is broadly consistent with, although toward the lower range of, estimates reported in previous melanoma studies. Danielsen et al. [21], in a systematic review and meta-analysis including 66 studies and 12,400 patients, reported pooled prevalence estimates ranging from 6% to 16% for depressive symptoms and from 7% to 30% for anxiety, depending on melanoma type and the timing of assessment [5]. A more recent meta-analysis by Kungwengwe et al. [7], including 7995 patients with cutaneous melanoma, demonstrated considerable variation over the disease trajectory, with anxiety and depression peaking during treatment at 30.6% and 18.4%, respectively, and declining thereafter [7]. The prevalence observed in our cohort therefore falls well within the range reported in the literature. Differences between studies are likely attributable, at least in part, to heterogeneity in patient populations, stage distribution, treatment status, timing of psychological assessment, and the screening instruments and cut-off values used.

The psychological burden observed in our cohort may also be considered in the context of epidemiological data from the general Polish population. In the nationwide EZOP II study, the lifetime prevalence of depressive episodes among Polish adults was estimated at 3.85%, while generalized anxiety disorder was reported in 1.16% of participants; lifetime panic attacks were reported by 7.04% [22]. In the present melanoma cohort, 8.8% of patients screened positive for clinically relevant depressive symptoms according to the PHQ-9, while 19.1% screened positive for clinically relevant anxiety symptoms according to the GAD-7. Although these findings suggest a considerable psychological burden among patients with melanoma, direct numerical comparisons with population-based prevalence estimates should be made with caution. EZOP II assessed psychiatric disorders using the WHO Composite International Diagnostic Interview (CIDI) [19], whereas the PHQ-9 and GAD-7 used in the present study are self-report screening instruments assessing current symptom burden rather than establishing formal psychiatric diagnoses. Moreover, the reference periods of the assessments differ. Nevertheless, placing our findings within the Polish epidemiological context further emphasizes the clinical relevance of depressive and anxiety symptoms in patients with melanoma. It is also important to notice that previous studies have suggested particularly high rates of depression among patients with lung, gynecological, and breast cancers, while in contrast, melanoma has generally been associated with a comparatively lower prevalence of depression [23,24,25]. Hartung et al. [25], for example, reported a prevalence of approximately 5% among patients with malignant melanoma, placing melanoma among cancer types with the lowest rates of depression.

One strength of the present study was the simultaneous use of two complementary measures for each psychological domain: PHQ-9 and HADS-D for depressive symptoms and GAD-7 and HADS-A for anxiety. These instruments should not be regarded as interchangeable. PHQ-9 directly reflects symptoms included in diagnostic criteria for depressive disorder and includes somatic manifestations, whereas HADS was specifically developed for populations with physical illness and was designed to reduce the contribution of somatic symptoms to psychological assessment [15,16,17,18,19].

Sex-related differences represent another important finding. Women had significantly higher PHQ-9 and GAD-7 scores than men, and the difference was also significant for HADS-A, whereas HADS-D scores did not differ significantly between sexes. The particularly consistent association between female sex and anxiety is in agreement with previous melanoma research. Kungwengwe et al. [7] found that female patients had approximately 80% higher odds of anxiety than males. Similarly, Beutel et al. [10], in a large cohort of 689 long-term melanoma survivors, reported higher psychological distress among women than men. More recent prospective evidence further supports this observation. Dugas-Breit et al. [9] followed 221 working-age patients during the first year after melanoma diagnosis and demonstrated poorer melanoma-specific HRQoL and a greater need for psycho-oncological support among women, particularly in earlier disease stages. Taken together, these observations suggest that sex may represent a clinically relevant marker of vulnerability to psychological distress in melanoma. The mechanisms underlying this association are likely multifactorial and cannot be determined from the present cross-sectional study.

Interestingly, neither depression nor anxiety was significantly associated with AJCC stage in our cohort. This finding deserves particular consideration, since it indicates that psychological burden is not necessarily proportional to objective oncological advancement. Previous evidence on this relationship has been inconsistent. The meta-analysis by Kungwengwe et al. [7] found greater depressive burden in patients with stage IV compared with stage I melanoma, suggesting that advanced disease may increase psychological vulnerability in some populations. Conversely, substantial psychological morbidity has also been documented in patients with localized melanoma. In a study of 386 newly diagnosed patients with stage IB–II disease, Beesley et al. [12] found anxiety in 32% and depression in 15%, together with considerable unmet supportive-care needs, particularly concerning disease progression and recurrence. Moreover, in the recent prospective study by Dugas-Breit et al. [9], impaired general well-being following diagnosis was observed independently of tumor stage, and psychological needs were particularly evident among women with relatively early-stage disease.

The absence of an association with melanoma stage in our study may therefore reflect the fact that emotional distress is shaped by factors extending well beyond objective tumor burden. Fear of recurrence, uncertainty regarding prognosis, coping strategies, perceived social support, personality characteristics, previous psychiatric morbidity, and individual interpretation of the cancer diagnosis may all contribute to psychological outcomes. This interpretation is supported by the long-term survivor study of Beutel et al. [11], in which lower social support, fear of recurrence, pessimism, and self-blame were consistently associated with greater distress and poorer QoL. Our finding that psychological scores were also unrelated to time since diagnosis further suggests that simple chronological measures may insufficiently reflect individual psychological adaptation. Longitudinal data indicate that melanoma-related distress may diminish rapidly in many patients after diagnosis and treatment, while a clinically relevant subgroup develops persistent or newly emerging symptoms over subsequent years [8]. Consequently, neither disease stage nor time elapsed since diagnosis should be used in isolation to determine which patients require psycho-oncological assessment.

A particularly important finding of the present study is the consistent relationship between psychological distress and dermatology-related QoL. Higher PHQ-9, HADS-D, GAD-7, and HADS-A scores were all significantly associated with higher DLQI scores. Although the cross-sectional design prevents determination of directionality, the consistency of these associations across two independent depression measures and two anxiety measures strengthens the observation that psychological well-being and patients’ everyday experience of melanoma are closely interconnected. Previous melanoma studies have also demonstrated relationships between psychological distress and QoL, although they have predominantly employed general oncology or melanoma-specific instruments. Beutel et al. [11] assessed melanoma survivors using PHQ-9 and GAD-7 together with the EORTC QLQ-C30 and found psychological distress to be closely related to poorer functioning and QoL. Beesley et al. [12] used the melanoma-specific Functional Assessment of Cancer Therapy–Melanoma (FACT-M) and likewise demonstrated psychological and QoL impairment among newly diagnosed patients. Dugas-Breit et al. [9] also used FACT-M and demonstrated substantial variability in QoL trajectories during the first year following diagnosis.

In contrast, the present study approached QoL from a dermatological perspective by using the DLQI. The DLQI evaluates the effect of skin disease on symptoms and feelings, everyday activities, leisure, work, interpersonal relationships, and treatment, thereby addressing domains that are particularly relevant to dermatological practice [20]. It is also one of the established instruments used for QoL assessment in skin cancer populations. In our targeted review of the available melanoma literature, we did not identify previous studies specifically examining the relationships between PHQ-9, HADS-D, GAD-7 and HADS-A scores and DLQI in a melanoma population. This should be interpreted cautiously as an observation based on the available literature rather than proof that no such data exist. Nevertheless, the present findings appear to add a distinct dermatology-oriented perspective to the established relationship between psychological distress and QoL in melanoma. They demonstrate that depression and anxiety are associated not only with general cancer-related or melanoma-specific HRQoL, but also with impairment captured by a widely used dermatological QoL instrument.

From a clinical perspective, these findings support the incorporation of psychological assessment into routine melanoma care. Importantly, screening should not be restricted to patients with advanced melanoma. Our results, together with previous studies, indicate that patients with early-stage disease may also experience substantial psychological distress. Brief validated instruments such as PHQ-9 and GAD-7 may facilitate the identification of individuals requiring further assessment, while positive screening results should be followed, where appropriate, by psychological or psychiatric evaluation. A multidisciplinary model involving dermatologists, oncologists, psycho-oncologists, psychologists, and psychiatrists may allow earlier recognition of clinically relevant distress and more individualized supportive care. Similar reasoning underpinned the clinical implications of our previous work in hidradenitis suppurativa, in which psychological screening and multidisciplinary care were emphasized as integral elements of comprehensive dermatological management.

Several strengths of the present study should be emphasized. Patients were consecutively recruited at two tertiary oncology centers, and the study population included individuals across the complete spectrum of AJCC stages, from melanoma in situ to metastatic disease. Psychological status was evaluated using multiple validated instruments, allowing complementary assessment of depressive and anxiety symptoms rather than reliance on a single screening tool. Moreover, the use of the DLQI provides a dermatology-specific perspective on QoL that has been relatively underexplored in relation to depression and anxiety among patients with melanoma.

Several limitations should nevertheless be acknowledged. First, the cross-sectional design precludes conclusions regarding causality or the direction of the observed associations. In particular, it cannot be determined whether depressive and anxiety symptoms contribute to poorer QoL, whether greater impairment of everyday functioning promotes psychological distress, or whether these relationships are bidirectional. Longitudinal assessment would be required to clarify the temporal dynamics of psychological symptoms and QoL throughout melanoma treatment and survivorship. This limitation is particularly relevant given previous evidence showing substantial changes in anxiety and depression over time following melanoma diagnosis [5]. Second, depression and anxiety were assessed using self-administered screening questionnaires without confirmatory structured psychiatric interviews. Although PHQ-9, GAD-7 and HADS are validated instruments, they identify symptom burden and patients at increased likelihood of psychological disorder rather than establishing formal psychiatric diagnoses. This may result in some degree of diagnostic misclassification and should be considered when interpreting prevalence estimates. Third, despite recruitment from two tertiary centers, the study was conducted exclusively in Poland with limited number of patients, and the findings may not be fully generalizable to populations with different healthcare systems, cultural backgrounds, socioeconomic conditions, or patterns of psycho-oncological support. In addition, several potentially relevant variables were not comprehensively assessed. These include educational level, marital and employment status, socioeconomic circumstances, non-melanoma comorbidities, previous psychiatric disorders and psychological or psychiatric treatment, psychotropic medication use, coping strategies, resilience, personality characteristics, perceived social support, and fear of cancer recurrence. Similar unmeasured variables were acknowledged in our previous analysis of illness acceptance conducted in the same melanoma cohort [13]. Fourth, detailed treatment-related variables were not included in the present analysis. Patients with melanoma may differ substantially with respect to recent surgery, systemic therapy, treatment-related adverse effects, disease recurrence, and whether they are undergoing active treatment or routine surveillance. Future studies should incorporate treatment modality and treatment phase when evaluating psychological outcomes. Fifth, although patients across all AJCC stages were included, individual stage subgroups were unequal and some were small, particularly stage 0. This may have reduced statistical power to detect subtle stage-related differences in psychological symptoms and should be considered when interpreting the absence of an association between AJCC stage and depression or anxiety. Finally, QoL was assessed using only one instrument, the DLQI. Although the DLQI is a well-established dermatology-specific measure and provides valuable information on the impact of disease on everyday functioning [20], it does not capture all dimensions that may be particularly relevant to patients with cancer or melanoma, such as fear of recurrence, uncertainty regarding prognosis, consequences of systemic anticancer therapy, or melanoma-specific concerns. Cancer-specific instruments such as the EORTC QLQ-C30 and melanoma-specific measures such as FACT-M could therefore have provided complementary information [26,27,28]. The absence of such instruments limits direct comparison of our QoL findings with much of the existing psycho-oncological melanoma literature.

At the same time, however, the exclusive use of DLQI may also be viewed as a distinctive aspect of the present study rather than solely as a limitation. Most previous studies examining depression, anxiety and QoL in melanoma have used general cancer-related or melanoma-specific instruments. By demonstrating consistent associations between four independent measures of psychological distress and DLQI, the present study provides evidence that depressive and anxiety symptoms are also closely related to the specifically dermatological dimensions of patients’ everyday functioning. This observation may be particularly relevant for dermatologists involved in long-term melanoma follow-up and warrants confirmation in future studies employing both dermatology-specific and melanoma- or cancer-specific QoL instruments simultaneously.

## 5. Conclusions

Depressive and anxiety symptoms represent a relevant component of the psychological burden experienced by patients with melanoma and are closely associated with poorer dermatology-related QoL. Female patients appear particularly vulnerable, especially to anxiety symptoms, whereas psychological distress was not clearly related to melanoma stage or time since diagnosis. These findings indicate that objective oncological characteristics alone may be insufficient to identify patients at risk of psychological difficulties. The consistent associations observed between depression, anxiety, and DLQI further emphasize the importance of considering psychological well-being as an integral component of melanoma care. Routine screening with brief validated instruments may facilitate early identification of patients requiring additional psychological or psycho-oncological support. Future longitudinal studies should clarify the direction of the relationship between psychological distress and QoL and identify independent predictors of impaired well-being throughout the melanoma disease trajectory.

## Figures and Tables

**Figure 1:**
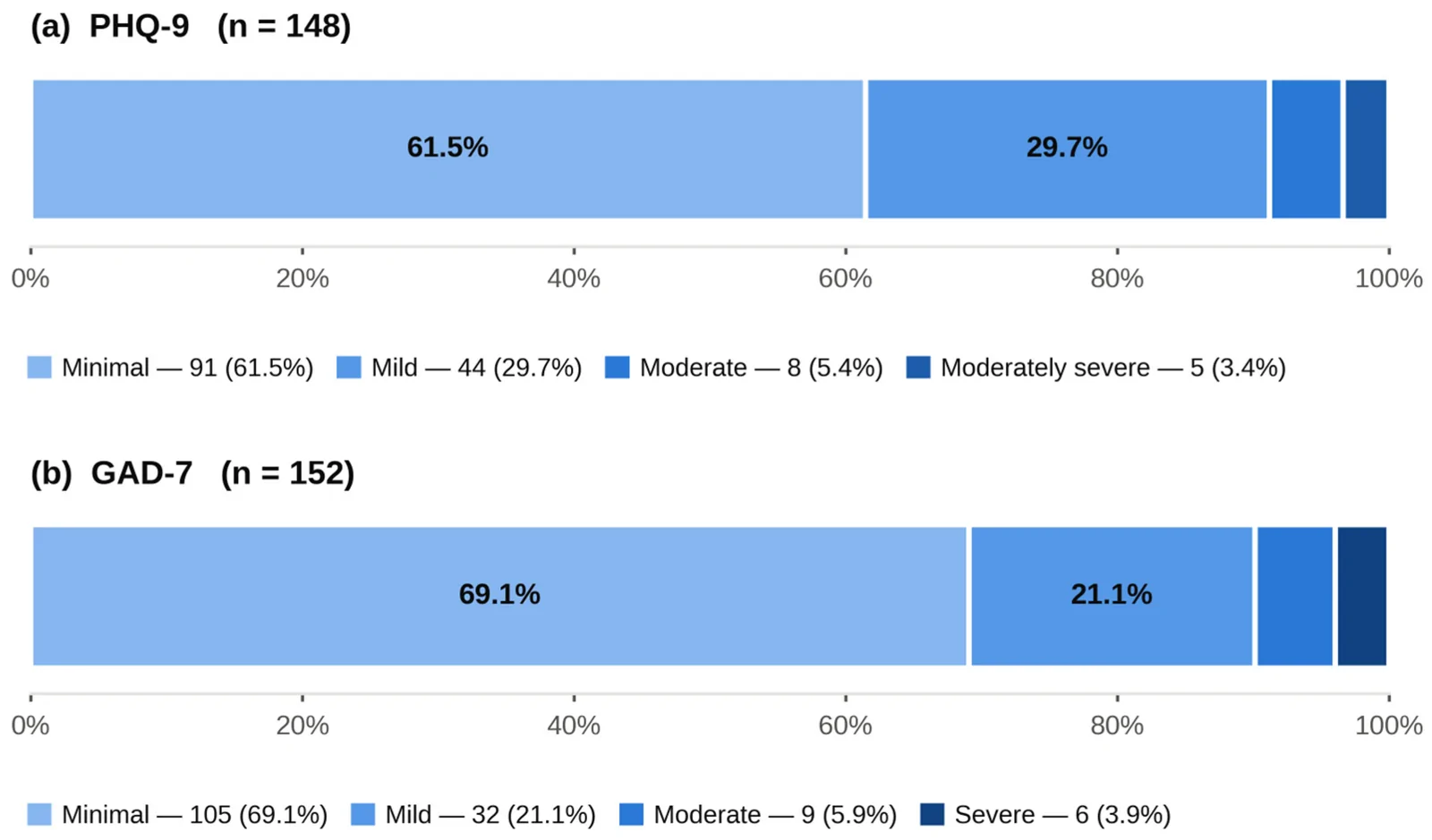
Depression (**a**) and anxiety (**b**) severity according to PHQ-9 (Patient Health Questionnaire-9) and GAD-7 (Generalized Anxiety Disorder-7), respectively.

**Figure 2:**
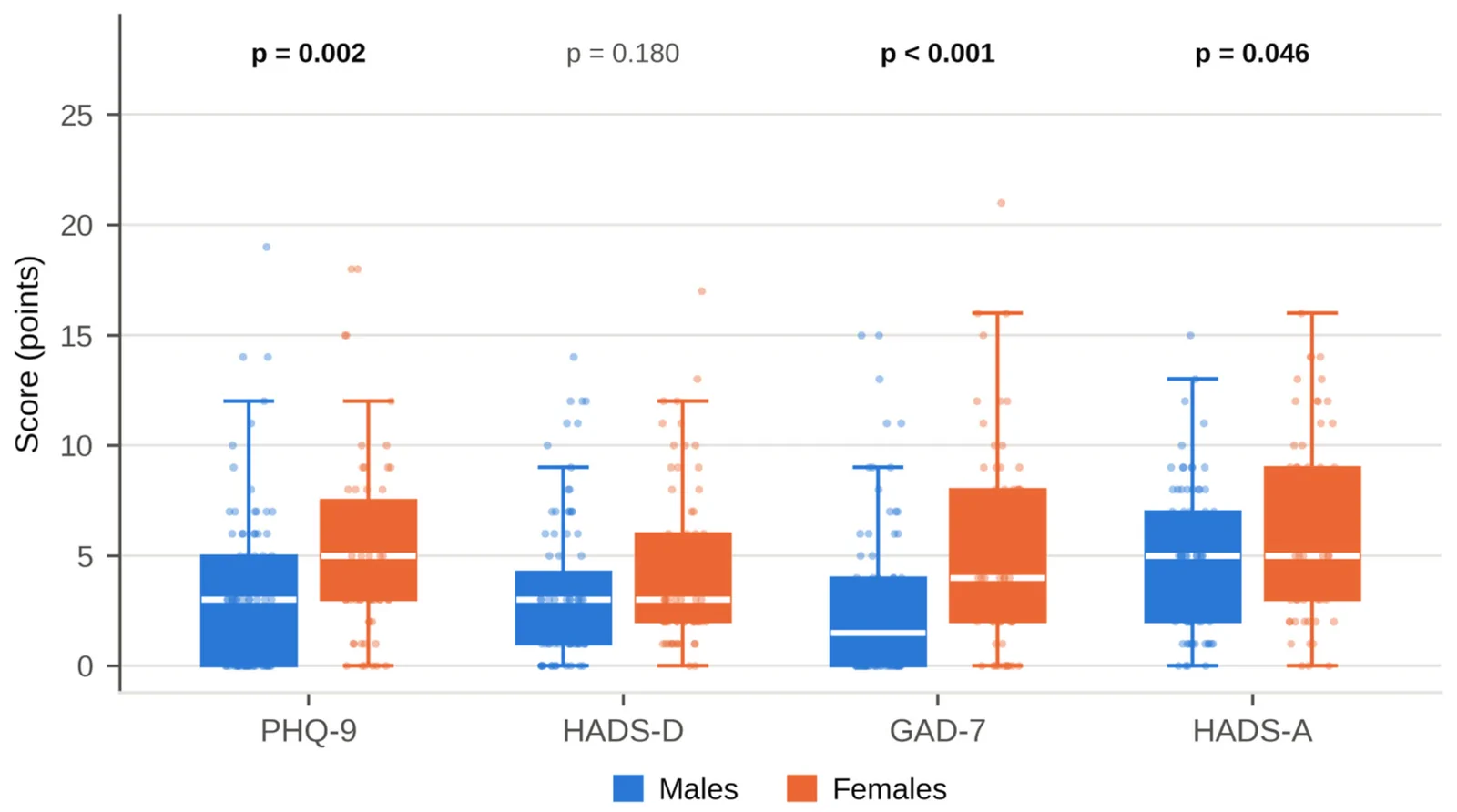
Depression and anxiety scores according to sex (PHQ-9—Patient Health Questionnaire-9, HADS-D—Hospital Anxiety and Depression Scale—Depression Subscale, GAD-7—Generalized Anxiety Disorder-7, HADS-A—Hospital Anxiety and Depression Scale, Anxiety Subscale).

**Figure 3:**
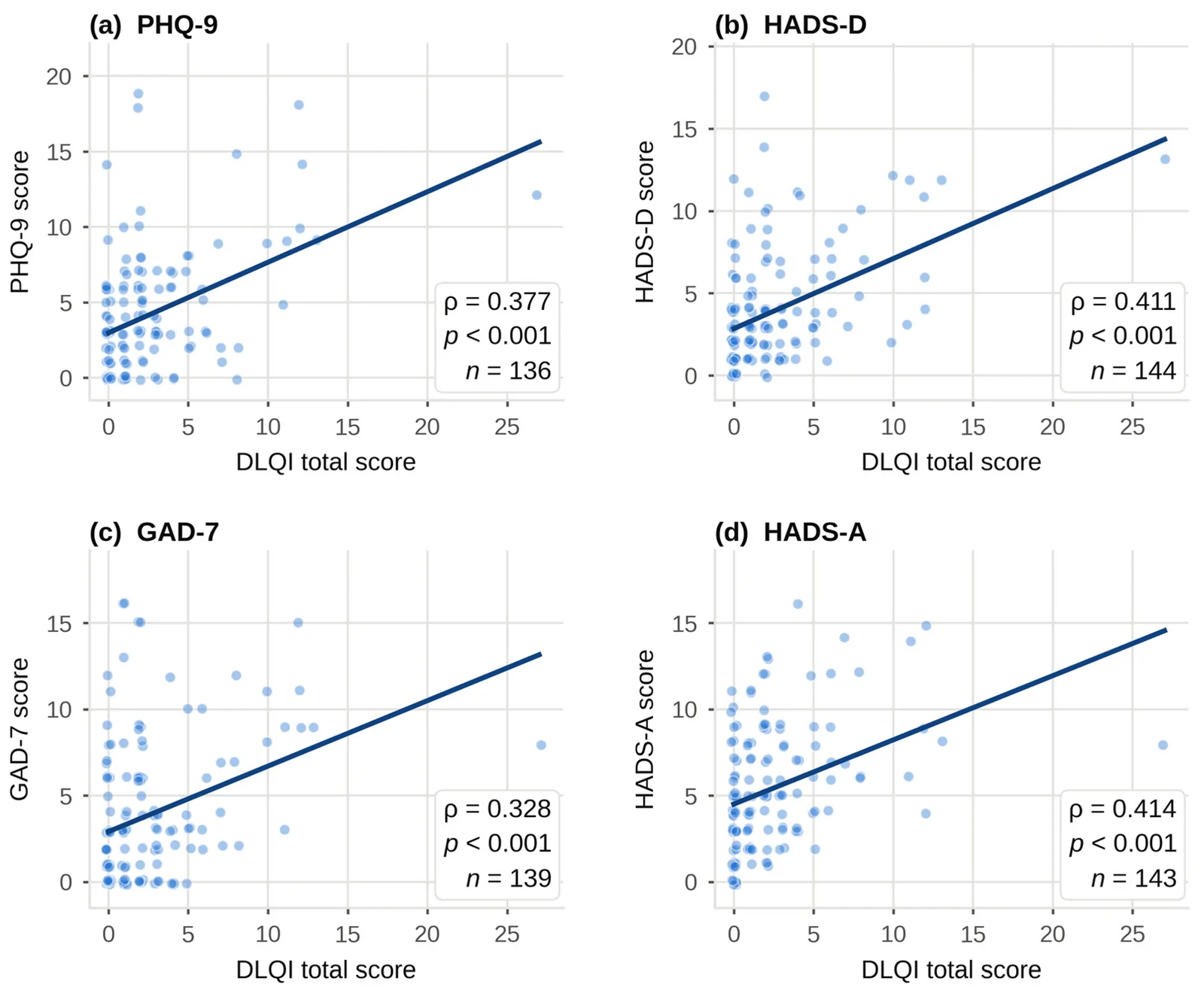
Correlations between depression and anxiety scores and quality of life measures (PHQ-9—Patient Health Questionnaire-9, HADS-D—Hospital Anxiety and Depression Scale—Depression Subscale, GAD-7—Generalized Anxiety Disorder-7, HADS-A-Hospital Anxiety and Depression Scale, Anxiety Subscale, DLQI—Dermatology Life Quality Index).

**Table 1:** Demographic and clinical characteristics of the study population (n = 162).

Characteristic	Value
Number of patients, n	162
Age, years, mean ± SD (n = 142)	59.8 ± 13.7
Age, years, median (IQR)	62 (50–71)
Sex, n (%)	
Female	67 (41.4)
Male	95 (58.6)
Time since melanoma diagnosis, weeks, median (IQR) (n = 149)	17 (9–48)
AJCC stage, n (%)	
Stage I	36 (22.2)
Stage II	47 (29.0)
Stage III	37 (22.8)
Stage IV	42 (25.9)

Data are presented as mean ± standard deviation (SD), median with interquartile range (IQR), or number (percentage), as appropriate. Percentages for scale-based variables are calculated using the number of participants with complete data as the denominator, which is given in the row label. AJCC—American Joint Committee on Cancer.

**Table 2:** Depression and anxiety scores in the overall study population and according to sex.

Measure	Whole Group	Males	Females	*p* Value
**Symptom severity, mean ± SD (n)**				
PHQ-9	4.16 ± 4.03 (148)	3.42 ± 3.69 (89)	5.29 ± 4.28 (59)	0.002
HADS-D	3.93 ± 3.47 (158)	3.58 ± 3.23 (92)	4.42 ± 3.76 (66)	0.180
GAD-7	3.84 ± 4.22 (152)	2.74 ± 3.50 (90)	5.44 ± 4.68 (62)	<0.001
HADS-A	5.43 ± 3.61 (157)	4.82 ± 3.12 (91)	6.26 ± 4.08 (66)	0.046
**Positive screen, n (%)**				
PHQ-9 ≥ 10	13 (8.8)	6 (6.7)	7 (11.9)	0.375
HADS-D ≥ 8	24 (15.2)	10 (10.9)	14 (21.2)	0.114
GAD-7 ≥ 8	29 (19.1)	9 (10.0)	20 (32.3)	0.001
HADS-A ≥ 8	43 (27.4)	19 (20.9)	24 (36.4)	0.046

Continuous variables were compared between males and females using the Mann–Whitney U test; proportions were compared using Fisher’s exact test. Percentages for positive screens are calculated within the corresponding sex using the number of participants with complete data for that instrument as the denominator. PHQ-9—Patient Health Questionnaire-9; HADS-D/HADS-A—Hospital Anxiety and Depression Scale, depression/anxiety subscale; GAD-7—Generalized Anxiety Disorder-7.

**Table 3:** Multivariable linear regression models with depressive and anxiety symptom scores as dependent variables.

Predictor	B	95% CI	β	*p* Value
**Dependent variable: PHQ-9 (n = 110; R = 0.592, R^2^ = 0.350, adjusted R^2^ = 0.319; F(5,104) = 11.21, *p* < 0.001)**				
DLQI total score	0.476	0.316 to 0.636	0.477	<0.001
Female sex	1.826	0.615 to 3.038	0.238	0.004
Age (per year)	−0.052	−0.095 to −0.008	−0.188	0.020
Time since diagnosis, log(weeks)	0.025	−0.523 to 0.573	0.008	0.928
AJCC stage (I–IV)	−0.045	−0.695 to 0.605	−0.013	0.891
**Dependent variable: HADS-D (n = 119; R = 0.525, R^2^ = 0.276, adjusted R^2^ = 0.244; F(5,113) = 8.62, *p* < 0.001)**				
DLQI total score	0.419	0.274 to 0.564	0.468	<0.001
Female sex	0.865	−0.197 to 1.928	0.129	0.110
Age (per year)	−0.028	−0.067 to 0.011	−0.116	0.152
Time since diagnosis, log(weeks)	−0.219	−0.704 to 0.265	−0.083	0.371
AJCC stage (I–IV)	0.253	−0.321 to 0.828	0.082	0.384
**Dependent variable: GAD-7 (n = 111; R = 0.529, R^2^ = 0.280, adjusted R^2^ = 0.245; F(5,105) = 8.15, *p* < 0.001)**				
DLQI total score	0.321	0.139 to 0.504	0.295	0.001
Female sex	2.263	0.891 to 3.636	0.272	0.002
Age (per year)	−0.090	−0.140 to −0.039	−0.296	0.001
Time since diagnosis, log(weeks)	0.162	−0.461 to 0.786	0.049	0.607
AJCC stage (I–IV)	−0.208	−0.941 to 0.526	−0.054	0.576
**Dependent variable: HADS-A (n = 119; R = 0.522, R^2^ = 0.272, adjusted R^2^ = 0.240; F(5,113) = 8.45, *p* < 0.001)**				
DLQI total score	0.376	0.217 to 0.536	0.383	<0.001
Female sex	1.154	−0.013 to 2.322	0.158	0.053
Age (per year)	−0.072	−0.115 to −0.029	−0.271	0.001
Time since diagnosis, log(weeks)	−0.296	−0.829 to 0.236	−0.103	0.273
AJCC stage (I–IV)	−0.320	−0.951 to 0.311	−0.094	0.317

B—unstandardised regression coefficient; β—standardised regression coefficient; CI—confidence interval; DLQI—Dermatology Life Quality Index; PHQ-9—Patient Health Questionnaire-9; HADS-D/HADS-A—Hospital Anxiety and Depression Scale, depression/anxiety subscale; GAD-7—Generalized Anxiety Disorder-7; AJCC—American Joint Committee on Cancer. All predictors were entered simultaneously. Models were fitted on complete cases.

## Data Availability

The datasets generated and/or analyzed during the current study are available from the corresponding author upon reasonable request. The datasets are not publicly available because they contain data derived from human participants and form part of an ongoing research project, for which additional analyses and publications are planned. Requests for access to the data will be considered on a case-by-case basis, subject to institutional approval, participant confidentiality, and applicable ethical and legal requirements.

## Abbreviations

The following abbreviations are used in this manuscript:

AJCC	American Join Committee on Cancer
DLQI	Dermatology Life Quality Index
GAD-7	Generalized Anxiety Disorder-7
HADS	Hospital Anxiety and Depression Scale
HADS-A	Hospital Anxiety and Depression Scale—Anxiety
HADS-D	Hospital Anxiety and Depression Scale—Depression
HR-QoL	Health-related quality of life
N	Number FF
PHQ-9	Patient Health Questionnaire-9
QoL	Quality of Life
SD	Standard Deviation
